# The Role of Cancer-Associated Fibroblasts and Tumor-Associated Macrophages in the Tumor Microenvironment and Their Impact on Ovarian Cancer Survival and Therapy

**DOI:** 10.3390/curroncol33010059

**Published:** 2026-01-19

**Authors:** Alena A. McQuarter, Joseph Cruz, Celina R. Yamauchi, Mariem Chouchen, Cody S. Carter, Tonya J. Webb, Salma Khan

**Affiliations:** 1 Center for Health Disparities, Loma Linda University, Loma Linda, CA 92350, USA; amcquarter@students.llu.edu (A.A.M.); josephcruz@llu.edu (J.C.); ryamauchi@llu.edu (C.R.Y.); 2 Research Laboratory of Biophysics and Medical Technologies, The Higher Institute of Medical Technologies, University of Tunis El Manar, Tunis 1002, Tunisia; chouchenemariem02@gmail.com; 3 Department of Pathology and Human Anatomy, Loma Linda University School of Medicine, Loma Linda, CA 92350, USA; cocarter@llu.edu; 4 Department of Microbiology and Immunology, University of Maryland, Baltimore, MD 20742, USA; twebb@som.umaryland.edu; 5 Basic Science Research, School of Medicine, Loma Linda University, Loma Linda, CA 92350, USA

**Keywords:** epithelial ovarian cancer, cancer-associated fibroblasts, tumor-associated macrophages, tumor microenvironment, extracellular matrix, stromal remodeling, single-cell sequencing, targeted therapies

## Abstract

Ovarian cancer is the deadliest gynecologic cancer, mainly because it is often diagnosed late and resists standard treatments. The tumor microenvironment (TME) plays a major role in disease progression and therapy failure. Two key components of the TME, cancer-associated fibroblasts (CAFs) and tumor-associated macrophages (TAMs), create conditions that facilitate tumor growth and immune evasion. CAFs are highly diverse and originate from sources like fibroblasts and stem cells. They support cancer by remodeling the extracellular matrix, promoting angiogenesis, and releasing cytokines and growth factors that aid tumor survival. TAMs, which are usually in an M2 state, also promote metastasis and suppress immune responses by secreting immunosuppressive molecules. Together, CAFs and TAMs interact with cancer cells to activate pathways such as the TGF-β, IL-6, and PI3K/AKT pathways, which drive resistance to therapy. New treatments aim to block these interactions by targeting CAFs and TAMs through depletion, reprogramming, or pathway inhibition, often combined with immunotherapy. Advances such as single-cell sequencing and spatial transcriptomics now enable more precise identification of CAF and TAM subtypes, enabling more targeted therapies. This review summarizes their roles in epithelial ovarian cancer and explores how targeting these cells could improve outcomes.

## Simple Summary

Ovarian cancer is one of the most lethal gynecologic malignancies due to its late diagnosis and resistance to conventional therapies. This review explores how two significant components of the tumor microenvironment, cancer-associated fibroblasts (CAFs) and tumor-associated macrophages (TAMs), contribute to ovarian cancer progression and treatment failure. We summarize how these cells promote tumor growth, immune evasion, and therapy resistance by remodeling the extracellular matrix and releasing pro-tumorigenic factors. We also highlight promising therapeutic strategies targeting CAFs and TAMs, including reprogramming approaches and pathway inhibitors. Understanding these mechanisms may guide future precision therapies and improve patient outcomes.

## 1. Introduction

**Figure 1:**
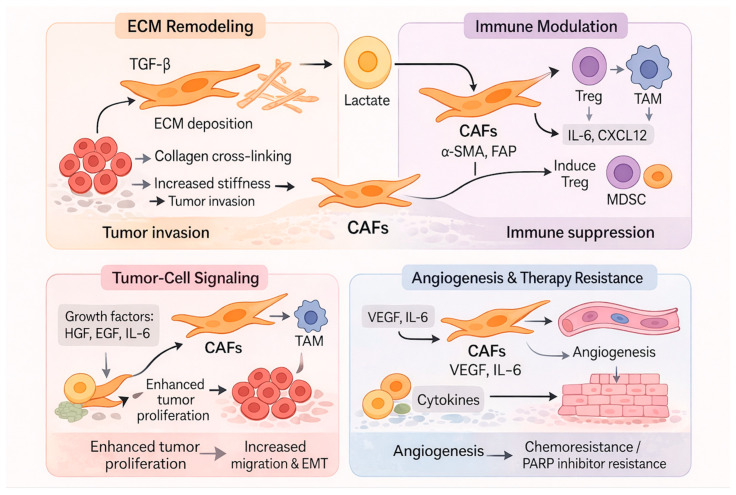
Signaling pathways mediated by cancer-associated fibroblasts (CAFs) and tumor-associated macrophages (TAMs) in ovarian cancer. CAFs secrete factors that engage multiple pathways in tumor cells, including transforming growth factor-beta (TGF-β)/suppressor of mothers against decapentaplegic (SMAD) (promoting epithelial-to-mesenchymal transition (EMT) and extracellular matrix (ECM) remodeling), and nuclear factor kappa-light-chain-enhancer of activated B cells (NF-κB) (driving inflammation and immune suppression. In tumor cells, CAF-derived signals activate downstream pathways in TAMs, including interleukin-6 (IL-6)/Janus kinase (JAK)/signal transducer and activator of transcription 3 (STAT3) (enhancing proliferation, angiogenesis, immune suppression, and chemoresistance) and phosphatidylinositol 3-kinase (PI3K)/protein kinase B (AKT)/mechanistic target of rapamycin kinase (mTOR) (conferring therapy resistance). Abbreviations: α-SMA, alpha-smooth muscle actin; FAP, fibroblast activation protein; Treg, regulatory T cell; CXCL12, C-X-C motif chemokine ligand 12; MDSCs, myeloid-derived suppressor cells; PARP, poly-ADP-ribose polymerase; HGF, hepatocyte growth factor; EGF, epidermal growth factor; VEGF, vascular endothelial growth factor. Created in Biorender. Alena A. McQuarter. (2026) https://BioRender.com (accessed on 4 January 2026).

Ovarian cancer is the most lethal gynecologic malignancy, mainly due to its asymptomatic onset and late-stage diagnosis, with over 70% of cases detected at advanced stages [1,2]. The predominant solid tumor phenotype in epithelial ovarian cancer (EOC) is highly aggressive and prone to peritoneal metastasis, often resulting in the accumulation of peritoneal fluid (ascites) in ovarian carcinoma patients. Standard treatment involves cytoreductive surgery followed by platinum-based chemotherapy, often combined with taxanes. Targeted therapies, such as poly-ADP-ribose polymerase (PARP) inhibitors and anti-angiogenic agents (e.g., bevacizumab), have modestly improved outcomes [3]. Although nearly 80% of patients initially respond, most relapse within three years, due to factors like drug resistance, tumor heterogeneity, impaired anti-tumor immunity, and adaptive changes in the tumor microenvironment (TME) [4]. Increasing evidence highlights the ovarian cancer TME, a dynamic ecosystem composed of cancer cells, stromal cells, immune cells, extracellular matrix (ECM), and soluble factors as a critical determinant of disease progression and treatment response [5,6]. These factors, along with limited effective screening and a lack of robust biomarkers, contribute to poor prognosis and the urgent need for novel, personalized therapeutic strategies [7]. Cancer-associated fibroblasts (CAFs) and tumor-associated macrophages (TAMs) comprise two central and dynamic cell types within the ovarian cancer TME that cooperate in complex ways to drive tumor progression and immune suppression, which is depicted in Figure 1.

CAFs originating from fibroblasts, stem cells, and epithelial cells undergo epithelial-to-mesenchymal transition (EMT) correlating with diversity in subtypes [8]. Through ECM remodeling and the secretion of cytokines and growth factors that promote angiogenesis and tumor cell survival, CAFs foster an immunosuppressive and pro-tumorigenic niche [9]. TAMs, which frequently represent the dominant immune cell population in the ovarian cancer TME, primarily arise from two sources: tissue-resident macrophages and bone marrow-derived infiltrating macrophages [10]. Similarly to CAFs, TAM programming and function is modulated by the assortment of cytokines, chemokines, metabolic cues, and interactions with cancer cells and other immune and stromal cell types [11,12].

In recent years, the substantial influence of CAFs and TAMs in immunosuppression, metastasis, and the development of therapy resistance in EOC has become more apparent. New treatments aim to re-educate the TME by targeting CAFs and TAMs through depletion, reprogramming, or pathway inhibition, often combined with immunotherapy [13]. Advances such as single-cell sequencing and spatial transcriptomics now enable more precise identification of CAF and TAM subtypes, enabling more targeted approaches [14]. This review summarizes their roles in EOC, and improving outcomes requires strategies that not only overcome therapeutic resistance but also enhance overall survival, which can be achieved through rational combination therapies such as pairing chemotherapy with PARP inhibitors or anti-angiogenic agents and integrating immunotherapy approaches like immune checkpoint blockade or adoptive T-cell transfer to reprogram the tumor microenvironment and sustain durable responses [15].

## 2. Materials and Methods

We searched the PubMed database, Google Scholar, and Google search engine for relevant peer-reviewed articles to include in this review. The search focused on keywords related to the tumor microenvironment (TME) and ovarian cancer, including “cancer-associated fibroblasts (CAFs),” “tumor-associated macrophages (TAMs),” “extracellular matrix (ECM),” “immune evasion,” “therapeutic resistance,” “stromal remodeling,” and “targeted therapies.” Article titles and abstracts were screened to remove irrelevant studies, and full-text articles were reviewed to determine inclusion in this paper. Studies were selected if they discussed the biological functions, signaling pathways, and therapeutic implications of CAFs and TAMs in the TME, with particular emphasis on ovarian cancer. Information from the selected articles was summarized to highlight key findings, mechanisms of action, clinical correlations, and potential therapeutic targets. Our methods were limited to full-text articles accessible through PubMed and Google searches; therefore, this review may not encompass studies published in other databases. Visual figures illustrating CAF and TAM signaling pathways and their interactions within the ovarian TME were created in BioRender (https://BioRender.com), and graphs were generated in Microsoft Word for visualization.

## 3. Cancer-Associated Fibroblasts in the Tumor Microenvironment

CAFs are a dominant and dynamic component of the TME, playing multifaceted roles in cancer progression, immune modulation, and therapy resistance [2]. Once considered passive structural elements, CAFs are now recognized as active participants in tumor biology, influencing nearly every aspect of cancer development and response to treatment [16]. Their functional diversity and plasticity present both challenges and opportunities for therapeutic intervention, particularly in EOC, where CAFs are deeply implicated in poor prognosis and resistance to therapy [17].

### 3.1. Origins and Heterogeneity of CAFs in Epithelial Ovarian Cancer

CAFs arise from diverse cellular sources, including resident fibroblasts, mesenchymal stem cells, pericytes, and epithelial cells undergoing EMT. This diversity contributes to their phenotypic and functional heterogeneity, which is a hallmark of CAF biology. Advanced technologies, such as single-cell RNA sequencing (scRNA-seq) and spatial transcriptomics, have revealed distinct CAF subpopulations with unique molecular profiles and spatial distributions within tumors [18,19]. Subtypes such as antigen-presenting CAFs (apCAFs) are characterized by high expression of major histocompatibility class II (MHC class II) genes; these genes play a crucial role in immune modulation and have a low or absent expression of alpha-smooth muscle actin (a-SMA). Inflammatory CAFs (iCAFs) secrete pro-inflammatory cytokines, such as interleukin-6 (IL-6) and C-X-C motif chemokine ligand 12 (CXCL12), which influence immune cell recruitment and promote tumor-promoting inflammation. In addition to these canonical subtypes, ECM-associated CAF populations have been identified in ovarian tumors and represent an important dimension of CAF heterogeneity not fully captured by the myofibroblastic CAF (myCAF)/iCAF/apCAF framework. These CAFs are enriched for genes encoding structural ECM components (e.g., collagens, fibronectin, periostin) and matrix-remodeling enzymes and are spatially localized to invasive tumor fronts and metastatic niches. Functionally, ECM-associated CAFs drive desmoplasia, enhance mechanical signaling, restrict immune cell infiltration, and contribute to therapy resistance by creating physical and biochemical barriers within the TME. Emerging evidence suggests that ECM-associated CAFs may exist along a functional continuum with myCAFs, shaped by factors such as transforming growth factor-beta (TGF-β) signaling, tissue stiffness, and tumor-derived cues [20].

**Figure 2:**
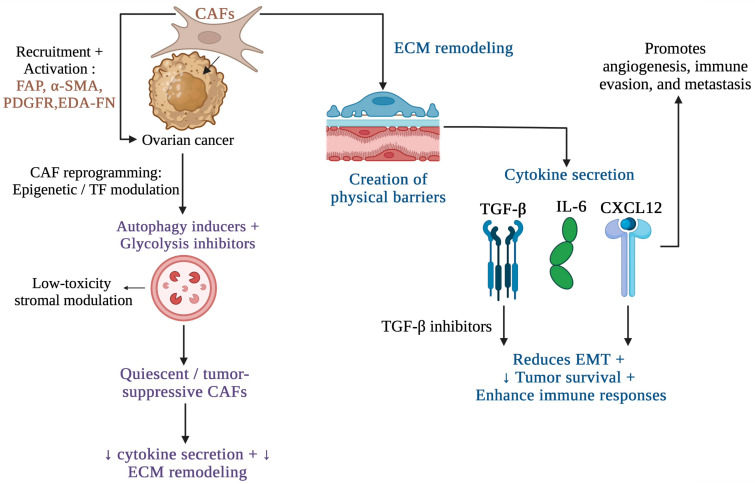
Cancer-associated fibroblasts (CAFs) in ovarian cancer remodel the extracellular matrix (ECM) and secrete cytokines such as transforming growth factor-beta (TGF-β) and interleukin-6 (IL-6), promoting immune evasion, epithelial-to-mesenchymal transition (EMT), and metastasis. Therapeutic strategies aim to reprogram CAFs into a quiescent state using autophagy inducers and glycolysis inhibitors, reducing cytokine secretion and ECM remodeling. Abbreviations: FAP, fibroblast activation protein; α-SMA, alpha-smooth muscle actin; PDGFR, platelet-derived growth factor receptor; EDA-FN, extra domain A-fibronectin; TF, transcription factor; CXCL12, C-X-C motif chemokine ligand 12; Created in Biorender. Mariem Chouchen. (2026) https://BioRender.com (accessed on 2 January 2026).

Collectively, CAF heterogeneity reflects significant functional plasticity, with CAF states dynamically shifting in response to microenvironmental signals. This adaptability complicates therapeutic targeting but also provides opportunities to disrupt key stromal programs that support tumor growth, immune evasion, and resistance to treatment. Understanding the origins, phenotypes, and context-dependent functions of CAF subtypes, including ECM-associated CAF populations, offers critical insight into their roles in ovarian cancer progression and highlights stromal pathways that may be exploited therapeutically. These CAF-mediated interactions and signaling pathways are summarized in Figure 2.

### 3.2. Functional Roles of CAFs in Tumor Progression in Epithelial Ovarian Cancer

CAFs contribute to tumor progression through several mechanisms, including ECM remodeling, angiogenesis, immune modulation, paracrine signaling, and support of cancer stem cells. CAFs produce and reorganize ECM components, creating a dense and stiff matrix that facilitates the invasion and metastasis of tumor cells. This altered ECM also acts as a physical barrier to immune cell infiltration and drug delivery [21]. By secreting pro-angiogenic factors such as vascular endothelial growth factor (VEGF), CAFs promote the formation of new blood vessels, ensuring a steady supply of nutrients and oxygen to the growing tumor. CAFs suppress antitumor immunity by secreting immunosuppressive cytokines (e.g., TGF-β, IL-6, interleukin-10 (IL-10)), recruiting regulatory immune cells, and forming physical barriers that exclude cytotoxic T cells. These actions contribute to immune evasion and resistance to immunotherapies. While most CAF subsets promote tumor progression, transcriptomic analyses further suggest that early-stage tumors retain fibroblast programs associated with tissue homeostasis and favorable prognosis, whereas advanced ovarian cancers are enriched for inflammatory and myCAF signatures linked to immune suppression and poor outcome in early stages [22,23,24]. CAFs engage in Bidirectional communication between tumor cells and CAFs, which is mediated through reciprocal signaling loops that evolve with disease stage and shape tumor progression, immune evasion, and therapy resistance. In high-grade serous ovarian carcinoma, tumor cells reprogram fibroblasts through TGF-β and inflammatory signaling, generating distinct CAF states that reciprocally promote tumor growth, ECM remodeling, and immune suppression via IL-6/signal transducer and activator of transcription 3 (STAT3) and CXCL12 signaling [22,25,26,27].

CAFs help maintain a niche that promotes cancer stem cells, which are implicated in recurrence and poor prognosis [28]. The functions of CAFs play a major role in the TME of ovarian cancer, including metastasis and immune suppression, but exploring the underlying cause for CAF tumor-progressive phenotypes is imperative as well.

### 3.3. Autophagy-Induced Ovarian Cancer Cells Promote Activation and Transformation of CAFs

Autophagy plays a critical role in fibroblast activation and CAF transformation within the ovarian cancer tumor microenvironment. Under physiological conditions, basal autophagy maintains fibroblast quiescence by limiting oxidative stress and regulating cellular metabolism; however, ovarian cancer-derived stressors such as hypoxia, reactive oxygen species, and TGF-β signaling disrupt this balance. Cancer cells induce autophagy in adjacent fibroblasts, driving metabolic reprogramming characterized by increased glycolysis, lactate secretion, and nutrient recycling that supports tumor growth and survival [29,30]. This autophagy-dependent rewiring promotes CAF phenoconversion, marked by elevated expression of α-SMA, collagen, fibronectin, and pro-tumorigenic cytokines, and reinforces extracellular matrix remodeling, immune suppression, and therapy resistance [31,32]. Dysregulated autophagy facilitates the establishment and maintenance of pro-tumorigenic CAF states in ovarian cancer, highlighting the need for cell-type-specific strategies when targeting autophagy within the tumor microenvironment.

### 3.4. CAF Plasticity and Transcriptional Regulation in Epithelial Ovarian Cancer

Fibroblast plasticity, the ability of fibroblasts to change phenotypes in response to environmental cues, is central to the formation and function of CAFs. In ovarian cancer, transcription factors such as transcription factor 21 (TCF21), cAMP response element binding protein 1 (CREB1), and suppressor of mothers against decapentaplegic homolog (SMAD2/3/4) have emerged as critical regulators of this process. TCF21 influences fibroblast differentiation and has been implicated in promoting a tumor-suppressive phenotype [33]. CREB1, involved in cellular stress responses, enhances CAF activation by regulating genes associated with inflammation and ECM remodeling. SMAD2/3/4 is downstream of TGF-β and regulates CAF differentiation, ECM remodeling, and immune exclusion. These factors drive the conversion of normal fibroblasts into CAFs, enabling them to remodel the ECM, secrete pro-tumorigenic cytokines, and adapt to the evolving tumor microenvironment [19]. Their plasticity enables dynamic adaptation to tumor signals, resulting in distinct phenotypes with varying functions.

Mapping transcriptional heterogeneity using scRNA-seq has revealed that CAFs in ovarian cancer are not a monolithic population. Instead, they comprise multiple subtypes with distinct gene expression profiles and functional roles. Understanding these transcriptional networks is essential for developing targeted therapies that modulate CAF behavior without disrupting normal tissue homeostasis [13,19].

### 3.5. CAF Risk Scores in Epithelial Ovarian Cancer

A comprehensive multi-omic analysis of EOC using data from The Cancer Genome Atlas has identified distinct CAF molecular subtypes, including myCAFs, iCAFs, and apCAFs, that are significantly associated with patient prognosis and response to immunotherapy [8]. Across these subtypes, elevated CAF risk scores consistently correlate with poor overall survival and increased infiltration of immunosuppressive immune populations, particularly macrophages and neutrophils. Mechanistically, high-risk CAF states are characterized by activation of TGF-β and IL-2/STAT signaling pathways, which contribute to immune evasion and tumor progression. Importantly, these CAF-associated risk signatures were validated in independent clinical cohorts, supporting their robustness as prognostic biomarkers along with others, including extra domain A-fibronectin (EDA-FN), fibroblasts activation protein (FAP), and platelet-derived growth factor receptor (PDGFR). EDA-FN is an alternatively spliced isoform of fibronectin that is selectively expressed in cancer-associated fibroblasts and tumor stroma, where it promotes extracellular matrix remodeling, tumor invasion, and immune evasion. FAP is a cell surface serine protease highly expressed on cancer-associated fibroblasts that promotes tumor progression by remodeling the extracellular matrix and suppressing anti-tumor immune responses. PDGFR is a receptor tyrosine kinase expressed on fibroblasts and other stromal cells that regulate cell proliferation, migration, and survival, and plays a key role in cancer-associated fibroblast activation and tumor–stroma interactions. Beyond survival prediction, CAF subtypes influence the overall composition and functional state of the TME by modulating the balance between pro-inflammatory and immunosuppressive signaling networks [34,35]. The development of CAF risk score models therefore provides a useful framework for patient stratification and for identifying individuals who may benefit from stromal-targeted or CAF-modulating therapeutic strategies. In ovarian cancer, high CAF risk scores have also been linked to resistance to immune checkpoint inhibitors (ICIs), highlighting CAFs as a major stromal barrier to effective immunotherapy. Integrating CAF risk stratification with treatment response data may enable correlation of established therapies with specific CAF expression patterns and subtypes, thereby informing rational combination strategies and guiding the development of novel microenvironment-directed interventions [36].

### 3.6. Potential Therapeutic Strategies Targeting CAFs in Epithelial Ovarian Cancer

**Figure 3:**
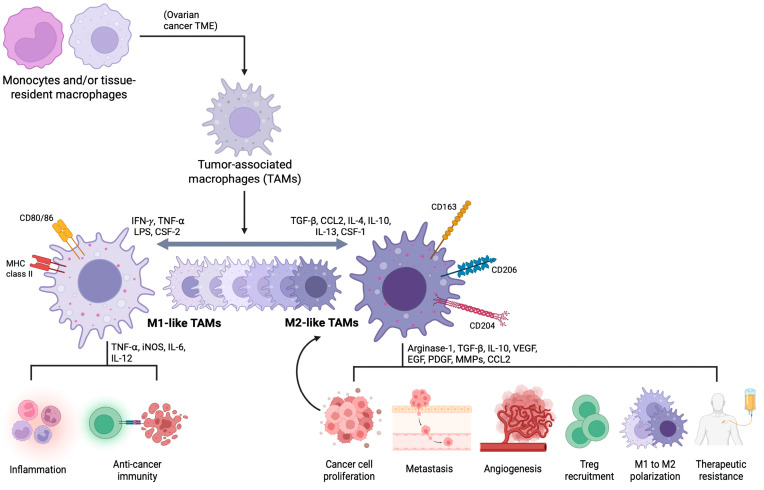
Tumor-associated macrophages (TAMs) in the ovarian cancer tumor microenvironment (TME) and their pathways. Infiltrating monocytes and tissue-resident macrophages give rise to TAMs in the tumor compartment. The assortment of cytokines, growth factors, and metabolic factors drives TAM polarization toward either the M1 or M2-like ends of the spectrum. Typically, TAMs with the M1 phenotype exert tumor-suppressing effects while those with the M2 phenotype play tumor-supportive roles. As highly heterogenous and plastic immune cells, individual TAMs may display mixed M1 and M2 expression profiles depending on where they reside, with the capacity to repolarize in response to changes in the TME; nevertheless, TAMs occupying the ovarian cancer niche are predominantly M2 skewed. Abbreviations: IFN-γ, interferon-gamma; TNF-α, tumor necrosis factor-alpha; LPS, lipopolysaccharide; CSF-2, granulocyte-macrophage colony-stimulating factor-2; iNOS, inducible nitric oxide synthase; IL-6, interleukin-6; IL-12; TGF-β, transforming growth factor-beta; CCL2, C-C motif chemokine ligand 2; CSF-1, colony-stimulating factor-1; VEGF, vascular endothelial growth factor; EGF, epidermal growth factor; PDGF, platelet-derived growth factor; MMPs, matrix metalloproteinases; Treg, regulatory T cell. Created in Biorender. Joseph Cruz. (2025) https://BioRender.com (accessed on 29 December 25).

Given their central role in tumor biology, CAFs have emerged as promising therapeutic targets. However, their heterogeneity and context-dependent functions pose significant challenges, including differential subtypes such as myCAFs, iCAFs, and apCAFs, which contribute uniquely to tumor biology [36]. This provides insight into strategies under investigation, including agents targeting TGF-β signaling, Hedgehog inhibitors, and phosphatidylinositol 3-kinase (PI3K)/protein kinase B (AKT) blockers, which have shown promise in preclinical models. Integrating stromal-targeted therapies with conventional chemotherapy and immunotherapy could overcome resistance and improve patient outcomes [2,36]. Multi-marker approaches and single-cell profiling are critical for distinguishing tumor-promoting from tumor-suppressive subsets. Biomarkers such as EDA-FN, FAP, and PDGFR are under evaluation for their diagnostic and therapeutic potential in ovarian cancer [21]. CAFs promote tumor progression, immune suppression, and treatment resistance by secreting factors such as IL-6 and activating TGF-β signaling, as depicted in Figure 3.

The current methods for treating ovarian cancer that specifically target CAFs include CAF depletion, CAF signaling inhibition, CAF reprogramming, metabolic interventions, combination therapies, and biomarker-driven precision medicine. Targeting surface markers such as FAP can selectively deplete tumor-promoting CAFs. However, indiscriminate depletion risks removing CAF subsets that may restrain tumor growth or support normal tissue homeostasis. Blocking key signaling pathways (e.g., TGF-β, IL-6, CXCL12) can disrupt the tumor-supporting effects of CAFs. For ovarian cancer, TGF-β inhibitors are particularly promising, as they can reduce EMT and enhance immune responses [37]. Reprogramming CAFs into a quiescent or tumor-suppressive state is a more nuanced approach. Agents that modulate epigenetic regulators or transcription factors involved in CAF activation are being explored to shift CAFs toward a less aggressive phenotype. Autophagy inducers and glycolysis inhibitors offer low-toxicity options for stromal modulation. Nutraceuticals, such as resveratrol, have shown potential in reversing CAF activation [38]. Integrating CAF-targeted therapies with conventional treatments (chemotherapy, radiotherapy) or immunotherapies may enhance overall efficacy. For example, combining CAF reprogramming agents with ICIs can restore immune surveillance and improve treatment outcomes [37,39,40]. Identifying reliable biomarkers to distinguish CAF subtypes is critical for personalized therapy. Advanced technologies, such as single-cell sequencing and spatial transcriptomics, are essential tools for mapping CAF populations and guiding targeted interventions [41].

One of the most clinically significant roles of CAFs is their contribution to therapy resistance. In ovarian cancer, CAFs have been shown to create physical barriers through ECM remodeling, which limits the effectiveness of chemotherapy and immunotherapy [42]. They can activate survival pathways in cancer cells via cytokines such as TGF-β and IL-6, thereby promoting EMT and increasing invasion. They can also suppress immune responses, reducing the efficacy of ICIs and other immunotherapies. These mechanisms make CAFs formidable obstacles to successful treatment. Their dynamic plasticity allows them to adapt to therapeutic pressures, further contributing to recurrence and poor outcomes [43]. Characteristic and treatment options for CAFs in epithelial ovarian cancer are highlighted in Table 1.

## 4. Tumor-Associated Macrophages (TAMs) in the Ovarian Cancer Tumor Microenvironment

Stromal and immune cells continuously interact to establish and maintain the complex, dynamic assortment of cellular and acellular components that is the ovarian cancer TME. In addition to CAFs, lymphocytes, granulocytes, dendritic cells, and endothelial cells, TAMs have emerged as key regulators of cancer biology. In many cases, they represent the most abundant immune cell population within the ovarian cancer TME, arising from infiltrating macrophages that originate from either bone marrow monocytes or tissue-resident macrophages derived from embryonic precursors [11,51]. TAM functional states are governed by the local cytokines, growth factors, and metabolic cues of the tumor site, where individual macrophages can polarize toward tumor-inhibiting M1 or tumor-promoting M2 phenotypes. A microenvironment rich in M1 TAMs facilitates a pro-inflammatory and anti-tumoral milieu. These macrophages actively present tumor-derived antigens to stimulate adaptive immune responses and preserve T-cell function. In addition, M1 TAMs perform tumoricidal roles through the production of oxidative species and by direct phagocytosis of tumor cells, while also limiting ECM remodeling and suppressing neovascularization [52]. Alternatively, the predominance of M2 TAMs advances tumor growth and perpetuates immunosuppression in the ovarian cancer microenvironment through multiple interconnected mechanisms. M2 macrophages exhibit defective antigen-processing and presentation machinery while simultaneously expressing a number of immune checkpoint proteins, obscuring tumor antigens and limiting immune surveillance. Additionally, M2 TAMs express a number of immune checkpoint proteins which suppress effector T-cell activity and facilitate cancer cell immune evasion [12]. Beyond immune dysfunction, M2 TAMs actively shape the composition of the TME by amplifying pro-vascularization and ECM remodeling pathways while simultaneously promoting growth, resistance to apoptosis, and cytoprotective signaling programs in malignant cells. Through extensive crosstalk with both cancerous and non-cancerous cells, M2 TAMs establish and sustain an immunosuppressive and tumor-supportive TME conducive to metastatic spread and resistance to chemo- and immunotherapies [52,53].

### 4.1. Characteristics of M1 and M2 TAMs in the TME in Ovarian Cancer

**Figure 4:**
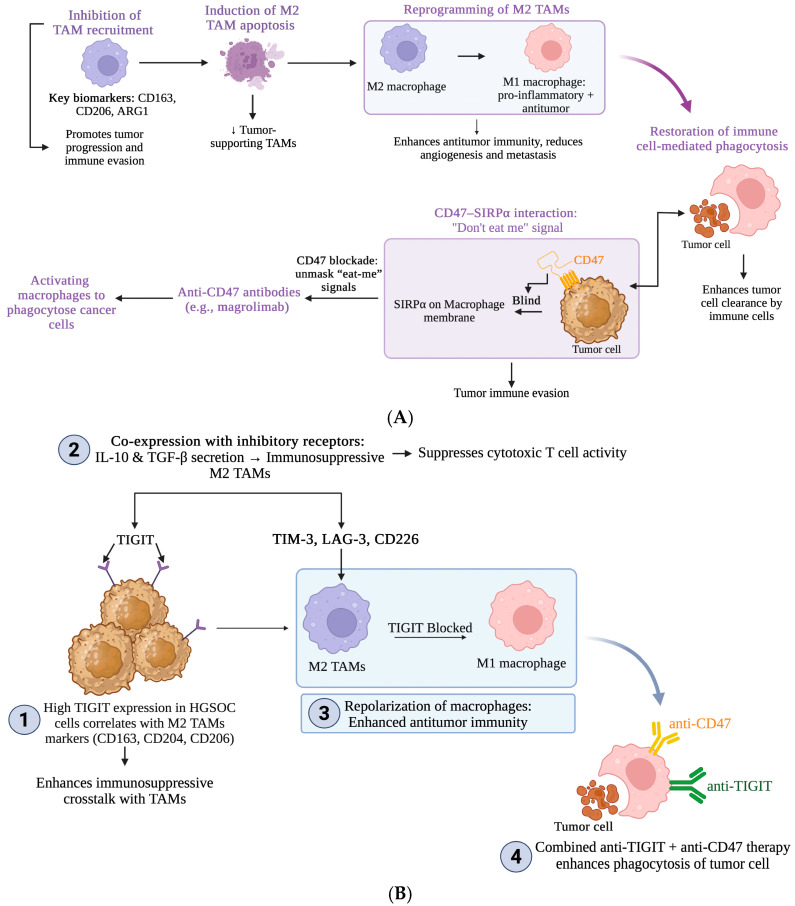
Treatment strategies to target tumor-associated macrophages (TAMs) in the tumor microenvironment of ovarian cancer. (**A**) Modulation of TAM recruitment and phagocytic activation in the TME. (**B**) Targeting TIGIT and CD47 pathways in M2 TAMs to enhance antitumor immunity. ARG-1, arginase-1; TIGIT, T-cell immunoreceptor with Ig and ITIM domain; LAG-3, lymphocyte-activation gene 3; TIM-3, T-cell immunoglobulin and mucin-domain containing-3. Created in Biorender. Mariem Chouchen. (2026) https://BioRender.com (accessed on 2 January 2026).

As illustrated in Figure 4, TAM populations in ovarian cancer are heterogenous and adaptable, acquiring M1 or M2-like characteristics according to their local TME. M1 macrophages, activated by tumor necrosis factor-alpha (TNF-α), interferon-gamma (IFN-γ), lipopolysaccharide (LPS), and granulocyte-macrophage colony-stimulating factor-2 (CSF-2), play pro-inflammatory and anti-tumorigenic roles through the expression of TNF-⍺, inducible nitric oxide synthase (iNOS), IL-12, C-X-C motif chemokine ligands 9/10/11 (CXCL9/10/11), and cell surface CD80/86 and MHC class II molecules. In this way, M1 TAMs enhance the activity and trafficking of effector T cells, promote Th1 immune responses, and facilitate tumor cell damage [54]. Conversely, M2 phenotype macrophages, induced by TGF-β, IL-4, IL-10, IL-13, colony-stimulating factor-1 (CSF-1), and C-C motif chemokine ligand 2 (CCL2), exert anti-inflammatory and tumor-supportive effects [13]. These M2 macrophages produce a diverse range of bioactive soluble and surface molecules, including arginase-1, IL-4, IL-10, IL-13, TGF-β, epidermal growth factor (EGF), VEGF, platelet-derived growth factor (PDGF), programmed death ligand-1 (PD-L1), CCL2, CD163, CD204, CD206, colony-stimulating factor-1 receptor (CSF-1R), and matrix metalloproteinases (MMPs). Functionally, these proteins accelerate tumor growth, induce EMT, stimulate angiogenesis, promote immune suppression, and foster therapeutic resistance [55]. Consequently, many studies have identified the abundance of M2 TAMs, along with their markers and products, as negative prognostic indicators in ovarian cancer.

### 4.2. M2 Phenotype Pathways in Ovarian Cancer

Although macrophage populations with mixed M1 and M2-like phenotypes have been isolated from ovarian cancer ascites and primary tumors, the majority exhibit robust M2-like phenotypes and expression profiles. Among the M2 phenotypic markers, CD163 is a monocyte and macrophage scavenger receptor, considered the most dominant M2 marker and highly specific to the M2 phenotype. Through CD163-mediated endocytosis, macrophages clear hemoglobin-haptoglobin complexes resulting from extravascular red blood cell accumulation, reducing reactive oxygen species buildup and upregulating the production of anti-inflammatory cytokines in the TME [56]. Its expression is associated with poor prognoses in a variety of malignancies, including EOC, where CD163-positive M2 TAMs also display the scavenger receptor CD204 and the mannose receptor-1 CD206 [57]. TAMs also play a complex role in therapeutic resistance and metastasis, facilitating crosstalk between cancerous and host-derived cells in the TME. Ovarian cancer cells secrete numerous factors into the tumor stroma that enhance macrophage localization and M2 polarization. For example, tumor cell-secreted CSF-1 promotes the influx of monocyte precursors expressing the CSF-1 receptor (CSF-1R) and promotes their differentiation into M2 TAMs. The CSF-1/CSF-1R pathway activates downstream signaling cascades, including PI3K/Akt, phospholipase C, MAPK, and ERK1/2, thereby driving cancer cell resistance to chemotherapeutic drugs. TAMs generate a variety of MMPs, such as MMP-9, an extracellular peptidase that facilitates degradation and remodeling of the ECM, which has been linked to the invasive capacity of ovarian cancer cells, while also improving the bioavailability of pro-angiogenic and tumor growth-promoting factors from the stroma [58]. The enzyme arginase-1, overexpressed by M2 macrophages, catabolizes arginine into ornithine and urea, which halts arginine-mediated cytotoxic T lymphocyte (CTL) differentiation and promotes T-cell starvation and exhaustion in the TME [59]. TGF-β is an immunosuppressive cytokine that plays roles in angiogenesis, extracellular matrix remodeling, tumor invasiveness, the recruitment of regulatory T cells (Treg) and macrophages, and the induction of the M2 phenotype. It is also believed to suppress polarization toward the M1 phenotype [60].

### 4.3. Potential Therapeutic Strategies Targeting TAMs in Ovarian Cancer

Therapeutic strategies being explored include the inhibition of TAM recruitment, induction of M2 TAM apoptosis, reprogramming of M2 TAMs to the M1 phenotype and restoration of antitumor functioning. Overall, each therapeutic approach has demonstrated greater efficacy than single-agent treatments.

In Table 2, we highlighted the origins of M2 TAMs, their phenotypic characteristics, and several treatment strategies under investigation, including M2 TAM depletion, recruitment inhibition, and M2-to-M1 reprogramming.

### 4.4. Inhibiting M2 TAM Recruitment in Epithelial Ovarian Cancer

Due to the central involvement of CSF-1/CSF-1R signaling in the attraction of TAMs to tumor sites and endorsement of M2 differentiation, CSF-1/CSF-1R targeting has become an appealing therapeutic strategy [66]. Preclinical studies have shown benefits from the use of CSF-1R inhibitors in combination with other therapies, including ICIs like anti-programmed cell death-1 (PD-1)/anti-PD-L1 antibodies to reduce the immunosuppressive effects of TAMs and improve T cell function, and with taxane drugs to eradicate tumor cells and simultaneously prevent TAM-induced chemoresistance [13,61,67,68]. Another strategy to reduce TAM infiltration involves blocking the CCL2/CCR2 axis, a pathway important for the migration and differentiation of M2-like TAMs within the EOC TME. CCR2 receptor inhibition in mouse models was observed to reduce TAM infiltration while improving the efficacy of paclitaxel and carboplatin chemotherapy [69,70]. In each case, approaches that aim to disrupt TAM trafficking prove most beneficial when used in combination with current anticancer treatments.

### 4.5. M2 TAM Reprogramming and Restoration of Antitumor Activity in Epithelial Ovarian Cancer

The highly plastic nature of TAMs permits repolarization between M2 and M1 activation states depending on the specific signals present in the TME. As shown in Figure 4A, CD47–SIRPα blockade is a strategy that aims to activate TAMs and other phagocytic cells against cancer cells. The cell surface protein CD47, a “don’t-eat-me” signal overexpressed on cancer cells and connected to worse prognosis, interacts with the signal regulatory protein alpha (SIRPα) on phagocytes to overpower pro-phagocytic “eat-me” signals and prevent engulfment [71]. By blocking this interaction, the anti-CD47 antibody magrolimab has been shown to unmask “eat-me” signals and induce macrophage-induced phagocytosis in cancer cell lines and ovarian cancer xenografts [62,72]. As a result, CD47–SIRPα blockade promotes TAM polarization toward the M1 phenotype [73]. Currently, however, clinical applications of CD47–SIRPα targeting focus on combination therapies. The study by Brauneck et al. (2023) examined T-cell immunoreceptor with Ig and ITIM domain (TIGIT) expression, its correlation with other M2-like TAM markers, and the effects of blocking both TIGIT and CD47 in EOC cells [57]. TIGIT expression, observed to be higher in more advanced and aggressive ovarian tumors, was correlated with CD163, CD204, and CD206. TIGIT was also more frequently co-expressed with the immune inhibitory receptors T-cell immunoglobulin mucin-3 (TIM-3), lymphocyte-activation gene 3 (LAG-3), and CD226 in the M2 phenotype. As shown in Figure 4B, blockade of TIGIT was shown to shift polarization from the M2 to M1 phenotype. Furthermore, anti-TIGIT in combination with anti-CD47 therapy enhanced in vitro phagocytosis of EOC cells [57]. A phase 1 trial (NCT05957536) is currently evaluating the anti-CD47/anti-HER2 bispecific antibody D3L-001 in advanced HER2-positive solid tumors [74]. Conversion of M2 TAMs to the M1 phenotype has also been attempted using TLR agonists to initiate the activation of immunostimulatory pathways and the production of pro-inflammatory cytokines. Many of these drugs, however, are being studied with specific and targeted mechanisms of delivery to reduce systemic toxicity [13,54]. Folate receptor beta (FRβ) is another key marker of immunosuppressive cells in the TME, including TAMs and myeloid-derived suppressor cells (MDSCs), making FRβ-targeted drugs a promising therapeutic strategy. Cresswell et al. (2020) found that targeting a folate-linked TLR7 agonist to TAMs and MDSCs in order to minimize off-target effects resulted in myeloid cell reprogramming, reduced immune suppression, and significantly increased CTL infiltration into the tumor site [75]. Existing evidence underscores the importance of M2-to-M1 TAM repolarization in restoring antitumor activity within the TME, particularly when combined with other targeted or chemotherapies.

### 4.6. Depleting M2 TAMs in Epithelial Ovarian Cancer

The use of bromodomain and extra-terminal domain (BET) inhibitors (BETi) has been identified as a potential strategy to target TAMs in ovarian tumors, particularly in combination with other approaches. BETi are small molecules that interfere with the function of BET epigenetic reader proteins, preventing them from activating immunosuppressive and carcinogenic expression profiles in cells of the TME [76]. Because TAMs have been found to confer resistance to anti-VEGF therapies, with high M2 TAM proportions notably strongly correlated with resistance, Wu et al. (2022) [64] examined whether reducing M2 density could help overcome resistance to anti-VEGF antibodies. In ovarian cancer cells and macrophage cell lines, BETi treatment selectively increased apoptosis in M2-like macrophages while also promoting M1 macrophage polarization. When used in combination, BETi and the anti-VEGF antibody bevacizumab suppressed tumor growth and TAM infiltration in ovarian cancer xenograft models [64,77]. The direct tumoricidal effects of BETi further strengthen its appeal as a treatment strategy. Wilson et al. (2018) [78] found that inhibition of BET proteins in ovarian cancer cell lines and patient-derived ovarian tumor xenografts led to synergistic effects with PARP inhibitors, impeding cancer cell growth, inducing DNA damage, and promoting apoptosis. Such combination therapies could make PARP inhibitors more appropriate for a wider variety of patients [78].

### 4.7. Potential Combination Therapies Targeting CAFs and TAMs in Epithelial Ovarian Cancer

TGF-β modulates both the expansion of stroma-remodeling CAF populations and the polarization of alternatively activated M2 TAMs in the ovarian TME. Upon recruitment and activation of fibroblasts at the tumor site, TGF-β signaling upregulates the expression of mesenchymal markers, favoring CAF phenotypes and transcription factors driving endothelial-to-mesenchymal transition. Once activated, CAFs contribute to TGF-β production, establishing a positive feedback loop that is propagated by promoting the protumoral TAM phenotype [79]. Attenuating TGF-β activity in combination with ICIs has gained popularity across a range of malignancies, with potential benefits for ovarian cancer [80]. Ultimately, the stimulatory effects of TGF-β on its production by CAFs and TAMs make TGF-β–targeted therapies an appealing strategy for restoring immune function and obstructing ovarian cancer growth; however, the application of TGF-β depletion to simultaneously modulate CAFs and TAMs is an area requiring further study. Another important molecule that triggers immunosuppression and promotes cancer cell survival in ovarian cancer is PD-L1. CAFs strongly induce PD-L1 in the TME, establishing a T-cell-suppressive network of stromal cells, further enhanced by CAF-produced cytokines [81]. PD-1/PD-L1 blockade, a popular immunotherapy under clinical development, could offer improved efficacy when combined with CAF- and TAM-focused treatments. A significant obstacle to existing ICIs remains the immunologically inactive, CTL-devoid status of advanced ovarian tumors, primarily due to the immunosuppressive fibroblasts and myeloid cells that comprise the TME [82]. Research has shown that TAMs represent the majority of PD-L1–expressing cells rather than cancer cells in advanced ovarian carcinomas [62], where they diminish antigen presentation and tumoricidal function of macrophages.

## 5. CAFs, TAMs, and Ascitic Fluid in Epithelial Ovarian Cancer

Ascitic fluid plays an active role in shaping CAF and TAM behavior within the EOC tumor TME, with direct consequences for disease progression, therapy resistance, and patient survival. Rather than a passive byproduct of disease, ovarian cancer ascites comprises a bioactive reservoir rich in cytokines (e.g., TGF-β, IL-6, IL-8), growth factors, lipids, ECM components, and extracellular vesicles, as well as diverse tumor, immune, and stromal cell populations [83]. The distinct peritoneal milieu supports tumor cell survival and carcinogenesis, while continuously re-educating CAFs and TAMs toward predominantly immunosuppressive, yet mutable phenotypes [84]. Persistent exposure to malignant ascites drives the activation and maintenance of pro-tumoral CAF phenotypes (e.g., myCAFs and ECM-associated CAFs). Ascites-derived TGF-β and mechanical cues promote α-SMA expression, collagen deposition, and matrix stiffening, while inflammatory mediators sustain cytokine-secreting iCAF states [85]. In turn, activated CAFs release an array of factors that promote immune exclusion by recruiting and polarizing pro-tumorigenic TAMs, restricting CTL infiltration, and constructing dense ECM barriers that limit drug penetration. Ascitic metabolites (e.g., lactate, fatty acids) further reprogram CAF metabolism, bolstering glycolysis and nutrient support for tumor cells, particularly within metastatic niches like the omentum [86]. Studies have shown that TAMs in this niche exhibit heterogeneous but predominantly M2-like phenotypes, producing immunosuppressive and tumor-supporting mediators, including TGF-β, IL-6, IL-10, CCL18, fibronectin, and tenascin C—molecules that favor tumor cell spheroid formation, migration, and the development of chemoresistance [87,88]. Ascites-mediated crosstalk between CAFs, TAMs, and tumor cells further enhances EMT, spheroid survival, and peritoneal dissemination, all hallmarks of advanced ovarian cancer. Clinically, high ascites volume and CAF/M2 TAM-rich TME signatures correlate with poor progression-free and overall survival, reflecting the self-reinforcing loop between ascitic signaling, stromal activation, and aggressive disease biology [89]. Collectively, these findings establish ascites as a critical regulator of CAF and TAM function in ovarian cancer and highlight ascites-associated CAF/TAM signaling pathways as invaluable targets to improve therapeutic efficacy and long-term patient outcomes.

## 6. Challenges and Future Goals

Despite significant advances in understanding the roles of CAFs and TAMs in ovarian cancer, major challenges remain that limit effective clinical translation. Foremost among these is the profound heterogeneity and plasticity of CAF and TAM subpopulations, which complicates their precise identification, functional annotation, and therapeutic targeting without disrupting tumor-restraining or homeostatic stromal functions. Additionally, dynamic crosstalk between CAFs, TAMs, tumor cells, and the extracellular matrix enables adaptive resistance to chemotherapy, targeted agents, and immunotherapy, often undermining single-pathway or single-cell–type interventions. Spatial and temporal variability within the tumor microenvironment further limits the predictive value of bulk biomarkers and preclinical models. Future goals, therefore center on integrating single-cell and spatial multi-omics with functional validation to define context-specific, targetable stromal and immune niches, develop robust CAF/TAM-based risk stratification tools, and guide rational combination therapies. Ultimately, translating these insights into biomarker-driven clinical trials that co-target tumor cells, CAFs, and TAMs will be essential to overcoming therapeutic resistance and improving survival outcomes for patients with ovarian cancer.

## 7. Conclusions

CAFs and TAMs are central architects of the ovarian cancer tumor microenvironment, shaping disease progression, immune evasion, and resistance to therapy. Rather than functioning as static cell populations, these stromal and immune compartments exhibit profound heterogeneity and plasticity, enabling tumors to adapt dynamically to therapeutic pressure and sustain malignant growth. From a therapeutic perspective, emerging evidence suggests that effective intervention will require moving beyond tumor cell-centric approaches toward strategies that disrupt pro-tumor stromal–immune circuits. Targeting immunosuppressive TAM phenotypes, modulating CAF-driven extracellular matrix remodeling, and interfering with key signaling axes such as PD-L1, TGF-β, and integrin-mediated pathways represent promising avenues to enhance immune responsiveness and improve treatment durability. Importantly, rational combination therapies that integrate stromal modulation with immunotherapy or conventional cytotoxic agents may overcome resistance mechanisms that have limited clinical benefit to date. Future efforts should prioritize high-resolution spatial and single-cell profiling to refine CAF and TAM subclassification, identify actionable niche-specific vulnerabilities, and guide patient stratification. Integrating these technologies with functional studies and biomarker-driven clinical trials will be essential to translate microenvironment-targeted strategies into meaningful survival benefits. Ultimately, reprogramming the tumor microenvironment—rather than merely eliminating tumor cells—offers a compelling framework for advancing precision therapy in ovarian cancer.

## Figures and Tables

**Table 1:** CAF-targeted therapeutic strategies in the tumor microenvironment of ovarian cancer.

Therapeutic Approach	Target Cell Population	Key Outcomes	Identified Weaknesses	Reference
TGF-β pathway inhibition (e.g., galunisertib, TGF-β neutralizing antibodies)	CAFs and CAF-driven ECM programs in EOC cell lines and xenograft models	Reduced CAF activation, decreased α-SMA expression and ECM deposition, improved immune infiltration, and enhanced chemotherapy sensitivity	Broad TGF-β inhibition may disrupt normal tissue homeostasis; context-dependent effects; limited ovarian-specific clinical trial data	[44,45]
FAP-targeted strategies (FAP inhibitors, FAP-CAR-T cells)	FAP^+^ CAFs in ovarian cancer xenograft and murine models	Selective depletion of pro-tumorigenic CAFs reduced tumor growth, enhanced T-cell infiltration, and improved response to immunotherapy	CAF heterogeneity limits complete stromal targeting; risk of on-target off-tumor toxicity; minimal clinical data in EOC	[46]
IL-6/JAK-STAT3 axis inhibition	Inflammatory CAFs (iCAFs) and tumor cells in EOC models	Decreased CAF-mediated cytokine signaling, reduced immune suppression, impaired tumor growth, and increased chemotherapy response	Redundant inflammatory signaling pathways; incomplete CAF reprogramming; systemic immune effects	[22]
ECM remodeling inhibition (LOX/LOXL inhibitors, collagen cross-linking inhibitors)	ECM-associated CAFs in ovarian cancer and peritoneal metastasis models	Reduced matrix stiffness, impaired invasion and metastatic spread, and improved drug penetration	Targeting ECM alone may not suppress inflammatory CAF signaling; limited ovarian-specific clinical validation	[47]
Autophagy modulation in CAFs	Metabolically active CAFs in EOC co-culture and xenograft models	Altered CAF metabolic support, reduced tumor growth, diminished therapy resistance, and impaired stromal support of cancer cells	Cell-type specificity remains challenging; autophagy inhibition may have opposing effects in cancer cells versus CAFs	[48]
Vitamin D receptor (VDR) activation	CAFs and stromal fibroblasts in ovarian and other epithelial tumor models	Reprogramming of CAFs toward a quiescent phenotype, reduced ECM production, enhanced immune infiltration	Variable VDR expression across CAF subsets; limited EOC-specific clinical trials	[49]
Hedgehog (Hh) pathway inhibition (e.g., vismodegib)	Stromal fibroblasts/CAFs in ovarian cancer models	Transient reduction in stromal density and CAF activation, modest improvement in chemotherapy delivery	Limited efficacy in clinical trials; compensatory stromal pathways; inconsistent outcomes	[50]

Abbreviations: TGF-β, transforming growth factor-beta; CAFs, cancer-associated fibroblasts; ECM, extracellular matrix; EOC, epithelial ovarian cancer; FAP, fibroblast activation protein; CAR-T cell, chimeric antigen receptor-T cell; IL-6, interleukin-6; JAK, Janus kinase; STAT3, signal transducer and activator of transcription 3; iCAFs, inflammatory cancer-associated fibroblasts; VDR, vitamin D receptor; Hh, hedgehog.

**Table 2:** Treatment strategies targeting M2 tumor-associated macrophages in the tumor microenvironment of ovarian cancer.

Therapeutic Approach	Target Cell Population	Key Outcomes	Identified Weaknesses	Reference
CSF-1R inhibition using pexidartinib (PLX3397) combined with paclitaxel	Phase Ib trial in advanced, treatment-refractory solid tumors, including EOCs	Of 38 patients, 3% had complete response, 13% had partial response, 34% stable disease, and 45% showed progressive disease	Omission of tumor biopsies—the effect of treatment on TAM and lymphocyte infiltration in the TME was not measured; some patients had a history of numerous failed therapies	[61]
Targeting anti-CD47–SIRPa axis using magrolimab (Hu-5F9) or TTI-621 combined with PARP inhibition	EOC cell lines, cell-line derived xenograft and patient-derived xenograft (PDX) mouse models	CD47 blockade enhanced the anti-tumor activity of PARP inhibitors and extended survival of the mice	Limited representation of human macrophage biology, immune activation, and tumor heterogeneity	[62]
Targeting CD206 TAMs with the Toll-like receptor 7/8 agonist imidazoquinoline IMDQ	Murine models with ovalbumin-expressing Lewis lung carcinoma (LLC-OVA) tumors	IMDQ led to reprogramming of pro-tumoral TAMs into anti-tumoral TAMs, increased T-cell response, and decreased tumor growth	Limited representation of human macrophage biology, immune activation, and tumor heterogeneity. Questionable duration of response	[63]
Treatment with the BET inhibitor ABBV-075 combined with the anti-VEGF antibody bevacizumab	EOC cell lines and murine xenograft models	BETi with bevacizumab induced cancer cell death and favored the survival and polarization of M1 TAMs. BETi sensitized EOC cells to anti-VEGF therapy.	Did not observe effects on other immune cell populations (e.g., effector T cells).	[64]
CAR-T cell-mediated depletion of FRβ-expressing TAMs	Murine ovarian cancer and colon cancer cell lines and xenograft models	Reduced M2-like TAMs and increased anti-tumor TAMs and T cells. Hindered tumor progression and prolonged survival in murine models.	Unknown duration and systemic impact of FRβ TAM depletion, due to the transient persistence of murine CAR-T cells. CAR-T cell therapies yield incomplete solid tumor response	[65]

Abbreviations: EOC, epithelial ovarian cancer; TAM, tumor-associated macrophages; TME, tumor microenvironment; CSF-1R, colony-stimulating factor-1 receptor; PARP, poly-ADP-ribose polymerase; BETi, bromodomain and extra-terminal domain inhibitor; VEGF, vascular endothelial growth factor; FRβ, folate receptor beta; CAR-T cell, chimeric antigen receptor T-cell.

## Data Availability

The original contributions (Figures and Tables) presented in this study are included in the article. No new data were created or analyzed in this study. Data sharing is not applicable to this article.

## Abbreviations

The following abbreviations are used in this manuscript:

CAFs	Cancer-associated fibroblasts
TME	Tumor microenvironment
TAMs	Tumor-associated macrophages
ECM	Extracellular matrix
EOC	Epithelial ovarian cancers
EMT	Epithelial-to-mesenchymal transition
iCAFs	Inflammatory cancer-associated fibroblasts
myCAFs	Myofibroblast cancer-associated fibroblasts
apCAFs	Antigen-presenting cancer-associated fibroblasts
ICIs	Immune checkpoint inhibitors
VEGF	Vascular endothelial growth factor
HGF	Hepatocyte growth factor
NF-κB	Nuclear factor kappa-light-chain-enhancer of activated B cells
FAP	Fibroblasts activation protein
EDA-FN	Extra domain A-fibronectin
TGF-β	Transforming growth factor-beta
PDGF	Platelet-derived growth factor
PDGFR	Platelet-derived growth factor receptor
IL-2	Interleukin-2
IL-4	Interleukin-4
IL-6	Interleukin-6
IL-10	Interleukin-10
IL-13	Interleukin-13
TCSF1	Cell survival factor 1
JAK	Janus kinase
STAT3	Signal transducer and activator of transcription 3
CXCL12	C-X-C motif chemokine ligand 12
CCL2	C-C motif chemokine ligand 2
CCR2	C-C motif chemokine ligand 2 receptor
ARG-1	Arginase-1
EGF	Epidermal growth factor
PD-1	Programmed cell death-1
PD-L1	Programmed death ligand-1
ROR2/PKC/CREB1	Components of the noncanonical Wnt signaling pathway
CREB1	cAMP response element binding protein 1
TCF21	Transcription factor 21
SMAD	suppressor of mothers against decapentaplegic
PI3K	Phosphatidylinositol 3-kinase
AKT	Protein kinase B
mTOR	Mechanistic target of rapamycin kinase
TIGIT	T-cell immunoreceptor with Ig and ITIM domains
CD47	Integration associated protein
SIRPa	Signal regulatory protein alpha
TIM-3	T-cell immunoglobulin and mucin-domain containing-3
LAG-3	Lymphocyte-activation gene 3
MDSCs	Myeloid-derived suppressor cells
Treg	Regulatory T cells
PARP	Poly-ADP-ribose polymerase
PARPi	Poly-ADP-ribose polymerase inhibitors
FRβ	Folate receptor beta
BET	Bromodomain and extra-terminal domain proteins
BETi	Bromodomain and extra-terminal domain inhibitors
CAR-T cell	Chimeric antigen receptor T-cell
CTL	Cytotoxic T-lymphocyte
TNF-α	Tumor necrosis factor alpha
IFN-y	Interferon-gamma
LPS	Lipopolysaccharide
CSF-1	Macrophage colony-stimulating factor-1
CSF-1R	Colony-stimulating factor-1 receptor
CSF-2	Granulocyte-macrophages colony-stimulating factor-2
α-SMA	Alpha-smooth muscle actin
